# Cerebral White Matter Myelination and Relations to Age, Gender, and Cognition: A Selective Review

**DOI:** 10.3389/fnhum.2021.662031

**Published:** 2021-07-06

**Authors:** Irina S. Buyanova, Marie Arsalidou

**Affiliations:** ^1^Neuropsy Lab, HSE University, Moscow, Russia; ^2^Center for Language and Brain, HSE University, Moscow, Russia; ^3^Cognitive Centre, Sirius University of Science and Technology, Sochi, Russia; ^4^Department of Psychology, York University, Toronto, ON, Canada

**Keywords:** white matter, myelination, fiber tracts, brain development, cognitive abilities, magnetic resonance imaging, diffusion tensor imaging

## Abstract

White matter makes up about fifty percent of the human brain. Maturation of white matter accompanies biological development and undergoes the most dramatic changes during childhood and adolescence. Despite the advances in neuroimaging techniques, controversy concerning spatial, and temporal patterns of myelination, as well as the degree to which the microstructural characteristics of white matter can vary in a healthy brain as a function of age, gender and cognitive abilities still exists. In a selective review we describe methods of assessing myelination and evaluate effects of age and gender in nine major fiber tracts, highlighting their role in higher-order cognitive functions. Our findings suggests that myelination indices vary by age, fiber tract, and hemisphere. Effects of gender were also identified, although some attribute differences to methodological factors or social and learning opportunities. Findings point to further directions of research that will improve our understanding of the complex myelination-behavior relation across development that may have implications for educational and clinical practice.

## Introduction

Humans can move, think and feel in part because their brain communicates signals through neurons. Neurons align themselves in brain layers such that gray matter consists of cell bodies and dendrites, and white matter (WM) consists of neuronal fibers with varying degree of myelination that allows transfer of signals across distributed brain areas. WM accounts for more than half of the total brain volume (Fields, 2010) and is involved in cognitive (Desmond, 2002; Ohlhauser et al., 2018), affective (Zhang et al., 2018), sensory (Pryweller et al., 2014; Chang et al., 2016), and motor functions (Sampaio-Baptista et al., 2013; Fleischman et al., 2015; Hollund et al., 2017). Myelination of cerebral WM changes with individual differences such as age (i.e., maturation) and learning (i.e., experience; Fields, 2010; Filley and Fields, 2016) and may be gender-specific (Passe et al., 1997; den Braber et al., 2013; Bourisly et al., 2017).

Myelination of neuronal axons in the gray matter (GM) of the brain is represented by radial and horizontal axons. Radial axonal fibers extend to the cytoarchitectonic Layer II and can pass long distances (up to 2 mm) connecting pyramidal neurons from the deeper layers with the cortical surface (Vogt and Vogt, 1919). Horizontal or tangential fibers are short-distance cortical axons, which originate mainly from inhibitory interneurons (Micheva et al., 2016) and lie in the two bands of Baillarger (1840) in cortical layers IV and V, in Exner’s stripe within the molecular Layer I (Stepanyants et al., 2009; Leuze et al., 2014), and late-myelinated *U*-fibers located within the superficial WM outside the GM (Leuze et al., 2014). Radial fibers can contain unmyelinated axons, which form temporary synaptic connections to local dendrites. Rate of myelination in short association fibers is region-specific (Flechsig, 1920), and is concurrent with the developmental trajectories of cognitive and motor skills (Turner, 2019).

The speed and timing of myelination of neuronal axons within association, projection, and commissural fiber tracts that comprise cerebral WM reflect their position in the functional hierarchy (Yakovlev and Lecours, 1967). For example, myelination of the motor root fibers is faster and shorter than that of sensory roots. In an adult brain, there are three myeloarchitectonic regions, which myelinate with different cycles as distinct tectogenetic units. A median periventricular zone comprises the median thalamus and the hypothalamus, the hippocampus, and the hippocampal rudiment and septal area. A paramedian (limbic) zone includes the paramedian thalamus, subthalamic region, the internal capsule, and the pallidum and striatum with the amygdala and claustrum. A supralimbic zone, including the bulk of the WM of the frontal, parietal, and temporal lobes and respective opercula.

Quantitative evaluation of WM volume and microstructural properties of myelinated fiber bundles is critical for understanding developmental trajectories in health and disease. Progressive myelination of WM pathways throughout infancy, childhood, and adolescence and into adulthood is concurrent with pronounced changes in cognitive abilities due to more rapid neural communication and integration of the information across functionally related brain regions (Giedd et al., 1999; Paus, 2010; Lebel and Beaulieu, 2011; Arshad et al., 2016; Morris et al., 2020).

White matter pathways in the brain are largely represented by association, commissural and projection fibers (Mandonnet et al., 2018). Association fibers connect close and distant brain areas within the same hemisphere and include the superior longitudinal fasciculus (SLF), arcuate fasciculus (AF), cingulum, inferior longitudinal fasciculus (ILF), inferior fronto-occipital fasciculus (IFOF), uncinate fasciculus (UF), middle longitudinal fasciculus (MdLF), and frontal aslant tract (FAT) (Catani and Thiebaut de Schotten, 2012; Gupta, 2017).

Commissural pathways connect the two hemispheres and are represented by the corpus callosum (CC), the anterior and posterior commissures, the hippocampal commissure, the habenular commissure, and the hypothalamic and cerebellar commissures (Catani and Thiebaut de Schotten, 2012; Gupta, 2017). Commissural pathways play a critical role in the integration and transfer of the information between the motor, perceptual, and cognitive modalities. Projection fibers pass through the internal capsule, corona radiata, cerebral peduncles, and brainstem to connect the cerebral cortex to other subcortical structures, such as spinal cord, deep cerebral nuclei, and brainstem nuclei, forming bidirectional (i.e., ascending and descending) connections. Output from the hippocampus to the cortex is formed by the fornix, which begins in the hippocampus and passes longitudinally toward the diencephalon and basal forebrain, arching over the thalamus (Catani and Thiebaut de Schotten, 2012; Gupta, 2017). Myelination cycles of the association pathways are protracted compared to commissural and projection fibers, as they connect cortical regions, providing functional coherence between brain areas.

Many studies focused on WM development (Lebel and Beaulieu, 2011; Sexton et al., 2014; Lebel and Deoni, 2018; Tamnes et al., 2018) and effects of WM lesions and cognitive impairments (Desmond, 2002; Valdés Hernández et al., 2013; Aso et al., 2017). Fewer studies investigate typical maturation of WM tracts in relation to cognitive functions. Literature associated with cerebral WM covers various pockets of knowledge such as methods of assessment, different conceptions of anatomical characteristics of fiber tracts, developmental effects, gender effects, and their association to mental function. Critically, a synthesis of the literature is lacking and aggregation of topics can help generate hypotheses for future research and create links between practice and research. Due to space limitations this review focuses on the most popular methods for assessing myelination, identifies anatomical considerations of nine fiber tracts, and presents current knowledge on developmental trajectories in relation to individual differences associated with age, gender, and cognition. Specifically, we cover eight major association tracts, SLF, AF, cingulum, ILF, IFOF, UF, MdLF, FAT; and one major commissural fiber system, the CC, which plays a critical role in the interhemispheric integration and transfer of the information. Although different methods exist that directly or indirectly assess microstructural properties of the WM, the most popular at this time is diffusion tensor imaging (DTI), thus, the review of individual differences related to age, gender and cognition focuses mainly on the DTI metrics, emphasizing its advantages, disadvantages, and research gaps for future studies.

## Methods for Assessing Myelination

The classic methods used for myelination assessment and investigation of WM was postmortem histological staining with hematoxylin/eosin (Flechsig, 1901; Yakovlev and Lecours, 1967), the Luxol fast blue technique (Bodhireddy et al., 1994), and subsequent immunohistochemical staining for myelin basic protein and myelin-associated glycoprotein (Itoyama et al., 1980). Critically, these methods cannot be used for *in vivo* studies. The development of non-invasive magnetic resonance imaging (MRI) techniques made it possible to indirectly assess myelination and other microstructural indices in the living brain. These methods include DTI, Myelin Water Imaging (MWI), and Magnetization Transfer Imaging (MTI), g-ratio imaging, and myelin mapping.

### Diffusion Tensor Imaging

Diffusion tensor imaging is one of the most popular noninvasive indirect imaging methods used to assess microstructure and orientation of myelinated WM fibers (Basser et al., 1994; Basser and Jones, 2002; Alexander et al., 2007). Diffusion MRI (dMRI) allows for investigation of the microstructure of the tissues by measuring diffusion of water molecules within the tissue considering their interaction with biological membranes and macromolecules and has been widely used to study individual differences in WM microstructure related to neurological and psychiatric conditions, as well as in healthy brains (Forkel et al., 2020).

Water diffusion can be characterized by the diffusion coefficient, ***D***, (mm^2^/s), which links diffusive flux and concentration gradient (Doran et al., 1996; Johansen-Berg and Behrens, 2009). Diffusion of water molecules in a solution is influenced by intermolecular interactions, temperature, molecular weight, and various active processes within the tissue (Beaulieu, 2002). Isotropic diffusion is described by a single apparent diffusion coefficient, which characterizes the interaction between the diffusing molecules and cellular structures (Basser and Jones, 2002; Beaulieu, 2002). In the brain’s WM, especially in highly myelinated regions, water diffusion is anisotropic (i.e., it is restricted by axonal membranes; Beaulieu, 2002) and can be described by a diffusion tensor, which replaces the scalar diffusion coefficient (Basser and Jones, 2002). The main direction of anisotropic diffusion within WM reflects fiber orientation, and, therefore, structural characteristics of a given brain region can be quantitatively determined by measuring water diffusion.

The displacement of the molecules is modeled using three-dimensional Gaussian distribution (O’Donnell and Westin, 2011). The diffusion tensor model is fitted using multi-linear regression; to estimate diffusion tensor, diffusion gradients must be applied at least along six noncollinear, noncoplanar directions (Basser et al., 1994). The diffusion tensor determines three orthogonal eigenvectors and associated eigenvalues (λ_1_, λ_2_, and λ_3_), which describe the diffusivity in the direction of each eigenvector. The direction of the main eigenvector corresponds to the direction of the fastest diffusion and determines the axis of a given fiber tract in the regions where the WM tracts do not branch and cross (Mori and Zhang, 2006; O’Donnell and Westin, 2011).

Diffusion tensor may be geometrically represented as an ellipsoid whose axes are of the length $\sqrt{2}\tau \lambda_{i}$ and are aligned with the eigenvectors (Basser et al., 1994). The eigenvalues of the diffusion tensor can be used to estimate widely used diffusion parameters, including (1) the mean diffusivity (MD), which reflects the total diffusion in a voxel, (2) longitudinal or axial diffusivity (AD), which is equal to the eigenvalue corresponding to the longest axis (λ_∥_λ_1_, assuming λ_1_≥λ_2_≥λ_3_≥0), (3) transverse or radial diffusivity (RD), which reflects the diffusion in the transverse plane and may indicate changes in axonal diameter, and (4) fractional anisotropy (FA), which characterizes the eccentricity of the diffusion ellipsoid and provides quantitative measure of the shape of the diffusion ranging from 0 to 1 (Basser and Jones, 2002; O’Donnell and Westin, 2011). MD is an inverse measure of membrane density and spacing between axons, which reflects the directionally invariant overall diffusion rate (Beaulieu, 2014). Notably, MD is similar in white and gray matter (Basser and Pierpaoli, 1996; Beaulieu, 2014). FA reflects the difference in the shape of the tensor ellipsoid between a perfect sphere and an ellipsoid that describes anisotropic diffusion.

**FIGURE 1:**
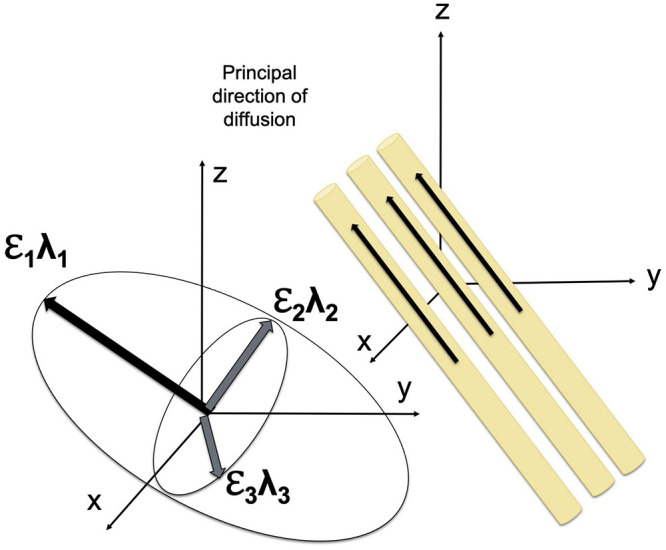
Diffusion tensor model. The orientation and shape of an ellipsoid describing water diffusion are characterized by three eigenvectors (ε_1_, ε_2_, and ε_3_) and eigenvalues (λ_1,_ λ_2,_ and λ_3_), respectively. The eigenvectors correspond to the principal axes of diffusion, and the eigenvalues reflect the diffusivities in three directions.

The diffusion of water molecules is measured using the pulsed-gradient, spin echo pulse sequence with a single-shot echo-planar imaging (Stejskal and Tanner, 1965; Westin et al., 2002; Alexander et al., 2007). Fiber orientation may be visualized using color-coding in which blue color corresponds to superior to inferior direction, red color reflects left to right orientation, and green denotes anterior to posterior direction (Pajevic and Pierpaoli, 1999). FA values are usually reflected by the brightness of the color (O’Donnell and Westin, 2011). Fiber trajectories are assessed using dMRI tractography (Basser et al., 2000), given that the orientation of the major eigenvector of the diffusion tensor is parallel to the fiber tracts (Figure 1). dMRI tractography starts at a “seed” point, that is a specified location at which the direction of the principal eigenvector is measured followed by moving a small fixed distance (≤1 mm) in that direction (tract integration). Evaluation of the fiber orientation is repeated and re-evaluated for successive small steps until the tract is terminated (Alexander et al., 2007). dMRI tractography has a number of limitations, including the susceptibility of estimates of eigenvector directions to thermal noise, physiologic fluctuations, and image artifacts. In addition, this method is based on the assessment of the principal eigenvector, thus making it impossible to study the direction of the fiber tracts that branch or cross (Alexander et al., 2007; O’Donnell and Westin, 2011). Nonetheless, this technique has important advantages for imaging brain structures *in vivo* and is widely used in normative and clinical studies. Advances in scanning sequences and analysis approaches make DTI one of the most popular methods for assessing WM microstructure and myelination.

### Myelin Water Imaging

Myelin Water Imaging is based on evaluation of myelin water fraction (MWF), which serves as a sensitive marker for myelin content. MWF corresponds to the fraction of water trapped by the myelin lipid bilayer (MacKay et al., 1994; Whittall et al., 1997; Deoni et al., 2012; MacKay and Laule, 2016) and allows for quantitative assessment of changes in fiber myelination (Deoni et al., 2011, 2012; Morris et al., 2020). MWI uses multi-exponential T_2_ relaxation data acquired with a multi-echo Gradient and Spin Echo sequence (MacKay and Laule, 2016; Faizy et al., 2020; Morris et al., 2020). The transverse relaxation time (T_2_, spin-spin relaxation time) determines the decay of transverse magnetization – the rate at which the spinning protons go out of phase with each other, i.e., lose phase coherence among the nuclei spinning perpendicularly to the main magnetic field B0. The transverse relaxation is related to the intrinsic field caused by the nearby protons. The individual nuclei within the tissue of interest precess in the transverse plane at slightly different rates so that their magnetic moments point to different directions and randomly interact with each other at the atomic or molecular level leading to the transverse decay of the MR signal and irreversible dephasing of the transverse magnetization. The exponential signal decay is therefore influenced by surrounding protons, and T_2_ for water trapped by the myelin sheath is shorter than T_2_ for extracellular or intracellular water, and decay amplitude is proportional to water amount in each medium.

Myelin Water Imaging is obtained from T_2_ spectrum after the extraction of exponential components in a T_2_ decay curve and is computed in each voxel as the ratio between the T_2_ distribution area corresponding to myelin water (T_2_ < 40–50 ms) to the total T_2_ distribution area, and thus is independent from the magnetic field. Fitting T_2_ decay with other T_2_ components allows for the extraction of the signal derived from myelin water with short T_2_ (Whittall et al., 1997; MacKay and Laule, 2016; Morris et al., 2020). At 3 Telsa, signal from myelin water has the shortest T_2_ component (< 40–50 ms; MacKay et al., 1994; Whittall et al., 1997; Deoni et al., 2012). This algorithm can also be applied to calculate T_2_^∗^ and T_1_ spectra (Hwang et al., 2004; Labadie et al., 2014).

The multicomponent model of relaxation signals evaluates the relative contributions of and interaction between multiple components, including intra- and extra-cellular water, cerebrospinal fluid, and myelin-related components (i.e., the water molecules within the myelin lipid bilayer; Lancaster et al., 2003; Deoni et al., 2013). However, to fit this model, a large number of measurements are needed leading to long acquisition times. Further, sophisticated preprocessing algorithms and long post-processing times impede the implementation of this method in pediatric imaging. Although new acquisition algorithms and processing strategies have been proposed, they still need to be validated before being introduced to routine clinical practice (Kulikova, 2015).

In agreement with postmortem data documenting myelination progress with age (Kaes, 1907), MWF increases in a caudo-rostral direction, following a parabolic trajectory with age. Changes in DTI indices usually exhibit linear association with age, and in studies which report non-linear relations peak age of myelination often depends on inclusion of young participants in selected WM regions (Westlye et al., 2010; Lebel et al., 2012). Indeed, peak age of myelination assessed by DTI indices is observed between 24 and 39 years (Sexton et al., 2014; Arshad et al., 2016), which is earlier than reported in MWI studies (Kochunov et al., 2012; Lebel et al., 2012) and MWF studies (Flynn et al., 2003; Arshad et al., 2016). The effect of age on DTI metrics is often different from that on MWF measures, showing weak or no correlations, as DTI may reflect other WM properties and components; therefore, some suggest that individual differences in myelination cannot be accurately described by variation in DTI-derived indices (Billiet et al., 2015; Arshad et al., 2016).

As evidenced by developmental studies, changes in MWF usually show quadratic association with age, as has also been indicated in postmortem studies (Arshad et al., 2016). Although DTI does show sensitivity to age-related changes in the cerebral WM, it may not be specific to myelin content (Jones et al., 2013a; Faizy et al., 2020). DTI metrics are significantly influenced by fiber density and orientation, as well as spatial organization (i.e., packing and crossing) and number of axons, whereas MWF reflects more closely myelin content, a hypothesis that has been validated histologically (Morris et al., 2020). However, the number of MWI studies investigating age-related changes in WM microstructure is limited (Dean et al., 2015; Arshad et al., 2016; Lynn et al., 2019), and recently developed MWI methods have not been validated with large normative samples and clinical settings. The computation of the MWF is also very sensitive to the acquisition parameters and computational settings, which makes it hard to compare results across studies.

### Magnetization Transfer Imaging

Magnetization transfer imaging is based on quantitative evaluation of magnetization exchange between spins via chemical exchange or cross-relaxation caused by spin diffusion (Edzes and Samulski, 1978; Fung, 1986). Spin diffusion is the quantum mechanical process by which spins can flip in a rigid soil medium and is associated with coupling between spin-lattice relaxations of different nuclei, such as macromolecular protons and water protons, in the presence of motion (Derome, 1987). Due to ultra-short transverse relaxation time T_2_ and low mobility of macromolecular protons associated with proteins and lipids, such as the galactocerebrosides within myelinated membranes (Kucharczyk et al., 1994; Engelbrecht et al., 1998), relaxation time of macromolecular spins cannot be directly visualized using MRI (Henkelman et al., 2001). Coupling between mobile water protons and protons associated with macromolecules results in exchange (i.e., magnetization transfer, MT), which allows for indirect imaging of these less mobile spins within the macromolecular proton fraction (Tozer et al., 2003).

The effect of cross relaxation in a heterogenous biological system is traditionally studied in the context of a two-pool model, which implies that a given biological system is comprised of two pools – the water protons (free pool), and protons associated with macromolecules (bound pool). These pools differ in their transverse relaxation times, with the transverse relaxation time of macromolecular protons and protons associated with biological membranes to be much smaller (< 1 ms) than that of water (> 10 ms). When two protons are close to each other, cross-relaxation between them may take place, resulting in simultaneous flips of spins via dipolar magnetic interaction. The spin-lattice relaxation in each pool is determined by a sum of two exponential decays characterized by two apparent relaxation rates.

The excitation of the proton spins bound to macromolecules by a radiofrequency pulse results in the transfer the energy to the nearby proton spins, which is referred to as the *magnetization transfer* effect (Henkelman et al., 2001). Traditionally, the MT effect has been assessed using an MT ratio (MTR), which measures a relative decrease in observed signal in the saturation experiment with an off-resonance radiofrequency pulse (Yarnykh, 2012) and allows for the evaluation of myelin content in the WM. Based on MT-weighted, T_1_-weighted, and proton-density weighted images, it is possible to build macromolecular proton fraction (MPF) maps, which describe the proportion of macromolecular protons contributing to MT effect as a result of cross relaxation with water protons (Yarnykh et al., 2018). MPF mapping is a new technique, which has been histologically validated and shows promise as a tool for accurate evaluation of fine changes in myelin content in fetal brain (Yarnykh and Yuan, 2004).

Magnetization transfer imaging is heavily dependent on experimental conditions, including scanner type and sequence characteristics (Tozer et al., 2003). In addition, due to the interaction with other physical parameters of a two-pool model, such as the longitudinal relaxation rate (R_1_ = 1/T_1_), which also correlates with myelin content and is sensitive to various physiological parameters, including iron and calcium levels, axon count and size, the association between MTR and myelin content in tissue is nonlinear (Henkelman et al., 2001). Further, it is argued that MTR is sensitive to macromolecules associated with other cellular components, such as cytoskeleton, and, therefore, a solution given by a two-pool seems to be insufficient to accurately access complex interactions between molecular pools in living tissue (Sled and Pike, 2001).

Throughout development, MTR gradually increases in the posterior-to-anterior direction (Rademacher et al., 1999). Myelination rate is faster in the projection and commissural than in the association fibers, as indicated by lower MTR values in the association areas at first month of age (Rademacher et al., 1999). In the occipital and frontal regions, MTR reaches peak by 13 and 16 months, respectively. MTR in the thalamus, splenium and genu of the CC reaches 95% of the adult value by 10, 18, and 19 months, respectively (Xydis et al., 2006). Throughout adulthood, MTR in the occipital and temporal regions slightly increases, remaining relatively stable in other areas (Mehta et al., 1995; Armstrong et al., 2004).

Macromolecular proton fraction in the fetal brain (18–38 weeks) is fivefold lower than in adult brain (Yarnykh et al., 2018). In the brainstem, cerebellum, and thalamus it shows positive association with gestational age (Yarnykh et al., 2018).

The values of R_1_, which is principally sensitive to macromolecular fraction and tissue volume (Mezer et al., 2013; Stüber et al., 2014), reach peak between ages 30 and 50 years, and between 70 and 80 values drop substantially to values comparable to 8-year-old values (Yeatman et al., 2014). Over an 80-year period, changes in R_1_ follow parabolic curve, indicating a symmetric pattern of age-related changes.

In an adult brain, R_1_ values vary considerably among tracts, and their mature R_1_ and the rate of R_1_ changes are different (Yeatman et al., 2014). For example, the age-related R_1_ growth and decline in in the cingulum and ILF are twice as higher than those of the corticospinal tract and anterior thalamic radiation. Unlike R_1_, the rate of change in diffusivity during development exceeds that of aging, and therefore diffusivity data, particularly MD, do not follow a symmetric parabolic pattern, but instead can be described by a Poisson curve, sharply decreasing throughout childhood and adulthood, and then slowly increasing in the third decade of life (Lebel et al., 2012).

Although indices associated with macromolecular content in tissues seem to provide a more accurate estimate of developmental gain and age-related decline in WM than diffusion metrics, MTI is now mostly used in clinical research, and, to date, there is not enough normative pediatric data to comprehensively describe WM changes throughout development.

### *g*-Ratio

A new method which allows for the assessment of relative myelination and conduction velocity of an axon is based on the evaluation of the *g*-ratio (i.e., the ratio of the inner to the outer diameter of the myelin sheath; Waxman, 1980). This method has been introduced in 2011 and was applied in full brain WM imaging in 2013 (Campbell et al., 2018). The *g*-ratio is quantitatively expressed as a function of the myelin volume fraction (MVF) and the axon volume fraction (AVF):

$$g=\sqrt{\frac{1}{1+MVF/AVF}}$$

Diffusion MRI methods are used to assess the relative AVF by measuring the shape of the displacement distribution of water molecules and evaluation of the intra-axonal volume fraction within the diffusion visible volume (Campbell et al., 2018). The most popular diffusion models, which can be used to estimate the relative size of cellular compartments include neurite orientation density and dispersion imaging (Zhang et al., 2012), composite hindered and restricted model of diffusion (Assaf and Basser, 2005), diffusion basis (Wang et al., 2011), restriction spectrum imaging (White et al., 2013), and other methods described in detail in Campbell et al. (2018).

Myelin volume fraction can be obtained from MT saturation index (Helms et al., 2009; Campbell et al., 2018), quantitative MT imaging parameters (Campbell et al., 2018), MTR (Yarnykh and Yuan, 2004), proton density imaging (Duval et al., 2017), single-point two-pool modeling (Yarnykh, 2012), quantitative multicomponent T_2_ (Oh et al., 2006) or T_2_^∗^ (Du et al., 2007) relaxation techniques quantifying the myelin water fraction, macromolecular pool size from quantitative MT (Yarnykh, 2002), and single-point two-pool and inhomogeneous MT (Varma et al., 2015). *g*-ratio shows considerable variation among different fiber pathways and its values are shown to be in agreement with *ex vivo* histological measures (Ellerbrock and Mohammadi, 2017). However, the accuracy of the *g*-ratio largely relies on the calibration procedure, since the models used to assess MVF do not consider a tissue model, as diffusion models do (Campbell et al., 2018). Selection of an appropriate calibration approach significantly influences the obtained *g*-ratio, and, therefore, affects repeatability and comparability of the data (Ellerbrock and Mohammadi, 2017).

Despite the high potential of the *g*-ratio for *in vivo* estimation of relative myelination and the application of this method in normative and clinical studies of white matter evaluation, it has not been yet introduced into routine research practice, and the data on *g*-ratio changes across development are still limited (Dean et al., 2016).

The developmental trajectory of the myelin *g*-ratio is still unknown. It is expected that *g*-ratio decreases over time, as axon growth rate exceeds speed of myelination. Only a few studies have examined age-related changes in *g*-ratio (Dean et al., 2016; Cercignani et al., 2017). Throughout infancy and early childhood, *g*-ratio logarithmically decreases, reaching theoretically predicted values (Dean et al., 2016). Age-related nearly-linear increase in *g*-ratio from 20 to 76 years of age has been demonstrated with no gender effect identified (Cercignani et al., 2017).

### Myelin Mapping

An alternative method for assessing cortical myelin content, which is highly generalizable across different scanners and pulse sequences, and, thus, allows for the comparison of the data across studies, is based on combining T_1_-weighted (T_1_w) and T_2_-weighted (T_2_w) MRI to map myelin content (Glasser and Van Essen, 2011). T_1_ and T_2_ relaxation times reflect the interaction between water and macromolecules in tissue (Miot-Noirault et al., 1997). T_1_ strongly depends on membrane composition (Turner, 2019), particularly on lipid content (i.e., cholesterol and cerebroside levels; Koenig, 1991). Image intensity (e.g., brightness of the T_1_w image) reflects the distribution of myelin-associated macromolecules, whereas T_2_ relaxation time is associated with the molecular exchange and water diffusion, such that the lower intensity of a T_2_w image corresponds to higher myelin content (Miot-Noirault et al., 1997; Barkovich, 2000).

The T_1_w/T_2_w index makes it possible to quantitatively assess spatial distribution of myelin, to eliminate MR-related image intensity bias, and to increase the contrast to noise ratio (Glasser et al., 2013, 2014). Fast scanning time makes this method valuable for pediatric imaging. However, due to high sensitivity to intensity scale inconsistencies across different datasets, this method requires strict calibration, which is currently a matter of research (Ganzetti et al., 2014).

In summary, various techniques have been used to assess maturation of WM tracts in the human brain. Considerably more research is needed to replicate and validate findings across methodologies, which will lead to optimized scanning protocols for healthy and clinical signatures of WM changes. As the majority of the literature evaluates maturation of WM tracts using DTI, in what follows we focus on this literature to review considerations related with anatomical characteristics of common fiber tracts and results associated to age, gender and cognition.

## Anatomical Characteristics of Common Fiber Tracts

### Superior Longitudinal Fasciculus and Arcuate Fasciculus

A major association WM tract that links the temporoparietal junction and parietal areas with the frontal lobe is the SLF. Three SLF branches connect the ipsilateral frontal and opercular areas to the superior parietal lobe (dorsal subdivision, SLF I), to the angular gyrus (middle branch, SLF II), and to the supramarginal gyrus (ventral subdivision, SLF III). They are comprised of the frontoparietal network of the human brain with the SLF I being symmetric between the two hemispheres, and the SLF II and SLF III demonstrating rightward lateralization (Thiebaut de Schotten et al., 2011a). The AF is the fourth subdivision of the SLF, which links the posterior and middle superior temporal gyrus with the ventrolateral prefrontal cortex and the posterior region of Broca’s area (pars opercularis, Brodmann’s area 44; Kamali et al., 2014; Webb, 2017). However, some authors suggest that the AF is distinct from the SLF, with the AF connecting the caudal part of the superior temporal area with the dorsal prefrontal region of the cerebral cortex and comprising of the long, anterior, and posterior segments (Schmahmann and Pandya, 2006; Catani and Thiebaut de Schotten, 2012), and the SLF linking frontal and parietal cortical areas (Thiebaut de Schotten et al., 2011b; Forkel et al., 2020), although the anterior segment of the AF and the SLF III overlap (Forkel et al., 2020). The branch of the SLF, which connects the temporal cortex to the inferior parietal cortex (temporoparietal subdivision, SLF TP) is considered as the fifth subcomponent (Catani et al., 2005; Makris et al., 2005; Zhang et al., 2010; Webb, 2017), although it rarely appears in dMRI tractography studies.

### Cingulum

The cingulum is an association WM tract which extends sagittally and connects the orbital frontal regions with the pole, passing along the dorsal surface of the CC down the temporal lobe (Bubb et al., 2018). This bundle is viewed as a part of the limbic system and considered one of the central components of Papez circuit (Papez, 1995), which constitutes bilateral WM pathways between the anterior thalamic nuclei and cingulate cortex, as well as the parahippocampal region and the cingulate cortex. A substantial portion of cingular association fibers that run across the sagittal plane form intracortical connections, linking the medial parts of the frontal, temporal, and parietal lobes (Yakovlev and Locke, 1961; Schmahmann and Pandya, 2006). dMRI tractography reveals three components of the cingulum: subgenual, retrosplenial or supracallosal, and ventral parahippocampal subdivisions (Jones et al., 2013b), which are characterized by different FA values. Based on current anatomical evidence (Vogt et al., 2005; Heilbronner and Haber, 2014), the cingulum can be divided into five subdivisions, including the subgenual, rostral dorsal (anterior cingulate), caudal dorsal (retrosplenial), and temporal (parahippocampal) subcomponents and midcingulate cortical area.

### Inferior Longitudinal Fasciculus

The ILF is a ventral association WM tract, which connects the anterior temporal lobe, including the superior, middle, inferior temporal, and fusiform gyri, to the lingual, cuneate, lateral-occipital and occipito-polar regions of the occipital cortex (Shinoura et al., 2007; Panesar et al., 2018). Connectivity pattern of the ILF is characterized by a significant leftward-dominance (Panesar et al., 2018) and ventral position.

### Inferior Fronto-Occipital Fasciculus

The IFOF is one of the major association fiber systems, which is recognized as a part of the dorsal visual stream (Schmahmann and Pandya, 2006). It links the occipital cortex, temporo-basal areas, and superior parietal lobe with the frontal regions, passing through the temporal lobe and insula (Martino et al., 2010) and crossing the SLF, AF, ILF, and MdLF (Wu et al., 2016). High-angular-resolution diffusion imaging shows that the IFOF fibers vary in the sites of origin. Most of the fibers originate from the lateral and medial orbital frontal cortices, frontal polar cortex, including the fronto-marginal gyrus and transverse frontopolar gyrus, superior frontal gyrus and inferior frontal gyri, including the pars opercularis, pars triangularis, and pars orbitalis, and from the middle frontal gyrus (Wu et al., 2016). Fibers of the posterior portion of the IFOF terminate in the pericalcarine (cuneus and lingual gyrus) and fusiform gyri and occipital region, including the inferior, middle, and superior occipital lobes, with some fibers terminating in the superior parietal lobe, angular and postcentral gyri (Wu et al., 2016). A comprehensive study by Wu et al. (2016) suggests that the IFOF has at least five subcomponents characterized by different connectivity patterns, albeit other researchers divide this fasciculus into three (Conner et al., 2018) or two subdivisions (Martino et al., 2010), or consider it as a whole tract (Rofes et al., 2014).

### Uncinate Fasciculus

One of the long-range association bidirectional monosynaptic WM pathways is the UF, which connects the anterior temporal lobes and amygdala to the lateral orbitofrontal cortex and the anterior portion of the prefrontal cortex (Von Der Heide et al., 2013).

Commissural pathways connect two hemispheres and are composed of the CC, the anterior and posterior commissures, the hippocampal commissure, the habenular commissure, and the hypothalamic and cerebellar commissures (Catani and Thiebaut de Schotten, 2012; Gupta, 2017). As it follows from fiber location, commissural pathways play a critical role in the integration and transfer of the information between the motor, perceptual, and cognitive modalities.

Projection fibers pass through the internal capsule, corona radiata, cerebral peduncles, and brainstem connect the cerebral cortex to other subcortical structures, such as spinal cord (SC), deep cerebral nuclei, and brainstem nuclei, forming bidirectional (ascending and descending) connections. Output from the hippocampus to the cortex is formed by the fornix which begins in the hippocampus and passes longitudinally toward the diencephalon and basal forebrain, arching over the thalamus (Catani and Thiebaut de Schotten, 2012; Gupta, 2017).

### Middle Longitudinal Fasciculus

Middle longitudinal fasciculus is an association fiber tract, which was originally identified in the rhesus monkey brain (Schmahmann and Pandya, 2006; Wang et al., 2013), where it links the caudal and rostral portions of the STG, the caudal cingulated gyrus and the middle portion of the parahippocampal gyrus with the upper sector of the STG (Schmahmann and Pandya, 2006).

Neither the classical (Ludwig and Klingler, 1956), nor the contemporary fiber microdissection studies (Wang et al., 2013) have described the anatomical structure of MdLF. The existence of the MdLF has been first documented in a DTI study by Makris et al. (2009). Although DTI does not allow for an accurate tracking of the MdLF origin and destination, especially in voxels containing crossing fibers (Wang et al., 2013), more studies demonstrate successful delineation and differentiation of the MdLF from other ventral and dorsal association fiber pathways (Menjot de Champfleur et al., 2013). Notably, human dMRI tractography and microdissection studies show prominent differences in MdLF anatomy between humans and monkeys. As such, the human MdLF was shown to link the STG with the superior parietal lobule and parietooccipital region, with only minor connections with the angular gyrus, unlike monkey brain (Wang et al., 2013).

### Frontal Aslant Tract

The FAT interconnects the pars opercularis and pars triangularis of the inferior frontal gyrus and the anterior insula with the supplementary motor area (SMA) and pre-SMA (Chernoff et al., 2018; La Corte et al., 2021), and it was first identified in 2007 by Aron et al. (2007) and then described and named in the studies of Catani et al. (2012) and Thiebaut de Schotten et al. (2012). In addition, it was also identified in monkey (Thiebaut de Schotten et al., 2012) and human postmortem dissection studies (Lawes et al., 2008).

Maturation of fiber tracts has been documented *in vitro* (e.g., Yakovlev and Lecours, 1967). In what follows we focus on *in vivo* studies that use DTI to evaluate typical development identifying consistencies and inconsistencies in reports related to age, gender and cognitive abilities.

## Myelination and Age

The process of myelination follows a complex topographical and temporal pattern across development as demonstrated by histological studies (Yakovlev and Lecours, 1967). Changes in myelination are proposed to be synchronized among functionally related fiber networks (Valk and Knaap, 2013). In DTI studies, WM maturation is traditionally examined by assessing the changes in several metrics, including AD and FA, which increase with age as myelin thickness and axonal diameter and density increase, and RD and MD, which are known to decrease with age (Tamnes et al., 2018).

Critically, there is no period in development when WM microstructure in the brain is static. During childhood, adolescence, and early adulthood, WM maturation is fast and dynamic and shows pronounced increase in fiber density and myelination as indicated by an increase in FA and decrease in MD and RD (Schmithorst et al., 2002; Westlye et al., 2010; Lebel et al., 2012; Sexton et al., 2014). During mid-adulthood, WM structure is relatively stable, and in late adulthood, changes in WM accelerate again, reflecting degenerative processes, as indicated by a sharp reduction of FA and an increase in MD and RD (Schmithorst et al., 2002; Lebel et al., 2012).

The speed of myelination in a given region during ontogenesis corresponds to the position of the system in the functional hierarchy. The first site of myelination is the SC. Here, myelination begins at 12–14 weeks of pregnancy (Choi, 1981). Myelination cycle of the motor root fibers is faster and shorter than that of sensory roots. The last site of myelination in adults is intracortical fibers of the cerebral cortex (Yakovlev and Lecours, 1967). Within the period between midgestation and the end of the second postnatal year, this process is characterized by the greatest rate and undergoes the most dramatic changes (Brody et al., 1987; Engelbrecht et al., 1998).

**FIGURE 2:**
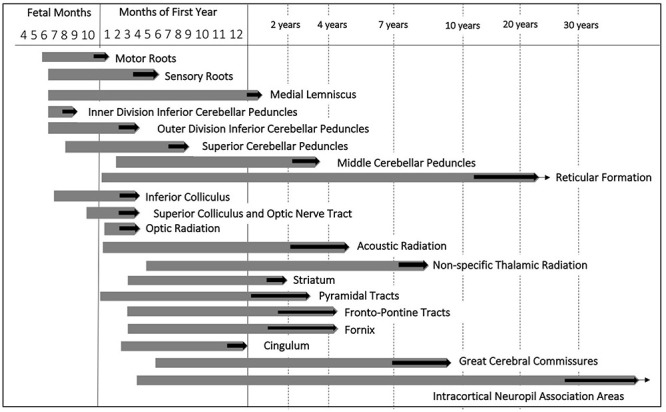
Myelination cycle adapted from Yakovlev and Lecours (1967). Chronological pattern of fiber myelination in the central nervous system. Thick black arrows indicate the approximate age range within which myelination of a given fiber tract terminates. Extending ends of the bars correspond to increasing staining intensity and density of myelinated fibers.

Myelination of the cranial nerve roots terminates early in the first postnatal year. The genu of the CC is myelinated by the 6th postnatal month. In the frontal, parietal and occipital lobes, the fibers are myelinated by 8–12 months of postnatal life (Barkovich and Kjos, 1988; Bird et al., 1989; Paus et al., 2001). In the central nervous system, sensory fibers which form the inputs to the thalamus and cerebral cortex myelinate earlier than fibers which participate in the integration of sensory information into movements (Bird et al., 1989; Paus et al., 2001). The longest myelination cycle is characteristic for the commissural and association fibers of the supralimbic zones. It exponentially continues to the years of maturity to senium (Yakovlev and Lecours, 1967). The development of the major fiber pathways from preterm age to the first postnatal age are summarized in Figure 2.

Myelination of the cerebral WM begins in the tenth fetal month. The most rapid growth of the brain occurs during the first 3 years of life (Dekaban, 1978). During childhood, an increase in WM volume exceeds that of the GM (Groeschel et al., 2010). A key common trend in WM development found across the majority of studies is its non-linear pattern across childhood and adolescence, accompanied by an overall increase in FA and a decrease in MD during the first years of life, which is more pronounced during early childhood (Krogsrud et al., 2016; Lebel et al., 2019). Fibers in the left hemisphere show greater maturation rate, and changes in myelination indices demonstrate an overall posterior-to-anterior gradient (Krogsrud et al., 2016). Interestingly, FA in the association tracts reaches its maximum 3–7 years before MD reaches minimum (Lebel et al., 2012). The transition from childhood to adolescence is accompanied by a pronounced increase in FA in the major fiber tracts, including the ILF, SLF, IFOF, fornix, cingulum, UF, and all subdivisions of the CC, indicating an increase in fiber density and myelination. Changes in MD show nonlinear pattern, reaching minimum between the age of 18–41 years and then gradually increasing (Lebel et al., 2012). Early development is characteristic for the CC and fornix, whose myelination is complete by the age of 20, whereas pathways which form frontal–temporal connections, including cingulum, UF, and SLF, demonstrate a more protracted maturation cycle, with the cingulum reaching peak FA values after 40 years (Lebel et al., 2012).

Myelination of the CC in children is incomplete (Brown et al., 2005), and with age it continues to mature, as it is one of the critical structures that sustains interhemispheric interaction (Banich and Brown, 2000). Importantly, different subdivisions of the CC demonstrate high degree of variability in size and maturation rates (Giedd et al., 1996). Posterior and mid regions of the CC show greater dependence on age compared to anterior portion of the tract, including the rostrum and genu of the CC that reach adult size in young children, thus exhibiting anterior to posterior gradient of myelination (Giedd et al., 1996). FA in the genu and splenium of the CC reaches peak values at 21 and 25 years, respectively, and in the body of the CC FA reaches maxima at the age of 35 years (Lebel et al., 2012). Critically, the assessed rate and temporal pattern of development of the splenium often vary by the age range of study participants and the fitting method used to model callosal maturation (Lebel et al., 2019).

Findings on age-related changes in other DTI metrics, such as RD, which reflects the rate of water diffusion in the direction perpendicular to the main axis of the axon, show that the most active maturation of association and projection pathways, which maintain cortical and brainstem integration, occurs during adolescence (Johansen-Berg and Behrens, 2009; Kumar et al., 2013). This is concurrent with the behavioral observations of pronounced improvements in reaction time that continue into late adolescence and adulthood (Asato et al., 2010; Arsalidou et al., 2013). Between 10 and 12 years of age, it is estimated that brain weight reaches adult values (Hedman et al., 2012). This period of WM maturation is accompanied by the maturation of critical cognitive functions, such as executive control and interhemispheric communication (Asato et al., 2010). The majority of fiber tracts, including the CC, SLF, ILF, IFOF, UF, and cingulum exhibit nonlinear developmental trajectory, as indicated by quadratic increases in FA and MD (Tamnes et al., 2018).

In the adult brain, the three myeloarchitectonic regions myelinate at different rates (Olson et al., 2015; Williamson and Lyons, 2018). Consistent with *in vitro* studies, DTI studies demonstrate that myelination of most association tracts continues into the third decade of life in a well-defined order (Olson et al., 2015; Williamson and Lyons, 2018). Also, age-related decrease in myelination in these fibers is delayed (Lebel et al., 2012). Myelination of the SC is mainly studied in animal and clinical studies (Tu et al., 2013; Antherieu et al., 2019; Mishra et al., 2020). Cervical SC microstructural properties are vulnerable to aging, as FA in this subdivision shows strong negative correlation with age (Agosta et al., 2007) and it dramatically decreases after the age of 55 years (Vedantam et al., 2013). Across the SC subdivisions, FA decreases in rostral-caudal direction, whereas MD remains relatively constant. The AD in the cervical subdivision is larger in thoracis and lumbar segments, indicating the presence of large-diameter myelinated axons (Vedantam et al., 2013). Notably, higher FA in the C2–C7 of the cervical SC predicts better performance in visuomotor tracking of precision grip force (Vedantam et al., 2013). The study of Lindberg et al. (2010) demonstrated asymmetry in WM density in the cervical SC: FA was higher in the lateral portion of the SC and on the right side, compared to the medial SC and the left side, respectively. Notably, across all cervical segments, only FA in the medial SC correlated with age, indicating higher sensitivity of this subdivision to aging.

During the transition from childhood (6–11 years) to adolescence (12–16 years), FA, fiber density and length in the SC increase, whereas MD decreases (Alizadeh et al., 2018). Findings on the association between other DTI metrics and age are controversial. Some studies demonstrate an increase in AD and MD with age (Reynolds et al., 2019), whereas others find an age-related decline in diffusivity measures (Singhi et al., 2012; Saksena et al., 2016).

Overall, finding on age-related changes in DTI indices in the SC are limited, and the association between SC myelination and cognitive and motor skill development is yet to be explored.

Critically, review studies that examine normative values across development, recognize that different methodological approaches such as preprocessing, statistical analyses and age group limits contribute to result differences among studies (Tamnes et al., 2018; Lebel et al., 2019). Specifically, they emphasize that linear models may not characterize the dynamic non-linear maturation patterns in myelination that occur across development and identify the need for improved methods (Tamnes et al., 2018; Lebel et al., 2019). Notably, dMRI tractography results are highly dependent on optimization algorithms (Harms et al., 2017). One of the most advanced algorithms is the gradient-free Powell conjugate-direction algorithm, which allows for considerable improvement of run time, parameter fit, and accuracy, whereas the parameter initialization approaches provide better fit for complex models (Harms et al., 2017).

## Myelination and Gender

Histological methods greatly contributed to the understanding of temporal pattern and regional specificity of the WM, however, data on gender differences in myelination pattern have been mostly obtained using MR-based techniques. Although male and female brains show many similarities, some studies highlight differences between the genders (e.g., Kanaan et al., 2012; Xin et al., 2019), yet others suggest that these differences are driven by environmental and cultural factors (Jäncke, 2018).

The proton density and T_2_-weighted MRI data corrected for total intracranial volume demonstrate that females exhibit a higher proportion of GM, while males are characterized by a greater percentage of total WM and cerebrospinal fluid (Gur et al., 1999), albeit some studies indicate that these differences are insignificant (Bourisly et al., 2017). Critically, the absolute differences in brain size and regional differences in the proportions of GM and WM between males and females mostly correspond to differences in WM volume (Passe et al., 1997; Allen et al., 2003; Schmithorst et al., 2008). In addition, after correcting for total cranial volume, no significant differences in the volumes of amygdala, hippocampus, and the dorsal prefrontal cortex between men and women are reported, although adult females are found to have significantly greater volume of orbitofrontal cortical region (Gur et al., 2002).

Several MRI studies demonstrate that the pattern of white and gray matter maturation in certain brain regions differs in males and females (De Bellis et al., 2001; den Braber et al., 2013; Ritchie et al., 2018). The total volumes of the cerebral gray and WM and CC show a pronounced sex-age interaction with males exhibiting a more significant age-related reduction of the gray matter and an increase in the WM volume and callosal region (De Bellis et al., 2001). According to the results of voxel-based morphometry analysis carried out by Bourisly et al. (2017), WM volume in the frontal, temporal, parietal, occipital, and insular regions of the brain is larger in men compared to women, and only WM of the postcentral gyrus in the right parietal lobe is greater in women compared to men. However, the authors of the study did not control for the total brain volume, although they claim that these differences cannot be explained by an assumption that the brain volume of men is, in general, bigger than that of women.

Data on gender differences in WM in frontal regions are contradictory. A study by Szeszko et al. (2003) reports that adult women have higher FA in the left frontal lobe compared to men. In addition, females exhibit a leftward frontal asymmetry in FA (i.e., higher FA values in the left hemisphere), which correlates with a better comprehension of verbal constructions and memory functioning in women, whereas males show no such asymmetry. These differences may be explained by the fact that maturation of the WM in the frontal lobes continues into late adulthood with a peak at about 45–50 years of age (Bartzokis et al., 2001; Schmithorst et al., 2008). A DTI study by Kanaan et al. (2012) found that adult males have higher FA values in the cerebellum and at the anterior portion of the left SLF, whereas females show greater FA values in the CC, interpreted to contribute to better interhemispheric communication and intellectual performance (Luders et al., 2007). Boys are also characterized by greater FA values in bilateral frontal WM, in the right AF, and in the left parietal and occipito-parietal WM. Interestingly, in the left frontal lobe, boys exhibit a positive and girls show a negative correlation between age and FA. In contrast, FA in the right AF positively correlates with age in girls and negatively in boys. In the right frontal lobe and right occipito-temporo-parietal WM girls exhibit a positive association between FA and age, whereas no significant correlation is found in boys (Schmithorst et al., 2008). An MRI study of healthy children and adolescents (6–17 years; Blanton et al., 2004) shows significant right > left asymmetry in the total cerebral volume, total cerebral WM, and WM of the middle and superior frontal gyri of boys, whereas girls are characterized by the same asymmetry pattern in the total cerebral WM and WM of the superior frontal gyrus. In addition, WM in the left inferior frontal gyrus shows age-dependent increase in boys, but not in girls. The authors speculate that these differences may be associated with gender effects in the development of speech and language lateralization.

The most rapid changes in the developmental pattern of the CC occur during infancy (Schmied et al., 2020) and findings on gender influence on the structure and development of the CC are inconsistent and largely depend on method and age when the microstructure was examined. One of the factors that may cause the discrepancies in findings on gender differences in the CC microstructure and development is the size of the brain: males, on average, are characterized by larger brain size than females, and therefore the size of the CC, if not adjusted for brain size, is also larger in males (Bishop and Wahlsten, 1997). Early data show no differences in the size and shape of the splenium of the CC between males and females, irrespective of the adjustment for brain size (Bishop and Wahlsten, 1997). However, recent DTI data on callosal area and thickness from infants (6–24 months) adjusted for brain size demonstrate a higher growth rate and size in boys than in girls, although the association between the microstructural characteristics and callosal size exhibits no significant gender differences (Schmied et al., 2020). Sullivan et al. (2010) show a significant gender-related difference in the development of the CC with age and their association with lower performance on cognitive and motor tasks observed in older adults. A DTI study by Schmithorst et al. (2008) shows that girls (mean age ∼12 years) have higher FA in the splenium of the CC than boys. Interestingly, the greater size of the splenium of the CC observed at autopsy was not confirmed by an early MRI study, which, reported a gender-related difference in minimum width of the callosal body and an age-related decline in anteroposterior distance and significant intraindividual variations in callosal size and shape regardless of age or gender (Byne et al., 1988).

Mechanisms that drive gender effects may rely on biological and experiential factors. Early studies suggest that differences in spatial and temporal pattern of myelination in boys and girls may be due to changes in sex steroid hormone levels during critical periods of development, accompanied by a 26-fold increase in testosterone and a 10-fold increase in estradiol level in males and females, respectively (Ducharme et al., 1976). Although steroid hormones are essential for myelination in both sexes, current research show that age-related changes and gender differences in sensitivity and responsiveness of brain structures to hormonal influences may explain different effect exerted by testosterone and estradiol on male and female brain at different stages of development (Patel et al., 2013).

Estradiol is known to affect hippocampal (Ducharme et al., 1976) and Schwann cell proliferation and differentiation (Chen et al., 2016), regulation of synaptic function, morphology, and plasticity (Smith et al., 2009), modulation of synaptogenesis and remodeling of neuronal circuits (Srivastava and Penzes, 2011; Sellers et al., 2015). Testosterone, in turn, stimulates neuronal growth at early stages of development, and both neuronal growth and myelogenesis at later stages (Stocker et al., 1994).

Normative values for DTI indices may differ across studies. Moreover, different imaging methods may provide different estimates of WM microstructure (Scholz et al., 2009). For example, some DTI studies show that the CC is more myelinated in males than in females (Westerhausen et al., 2003; Shin et al., 2005), whereas T_1_-weighted imaging demonstrates and opposite pattern (Scholz et al., 2009). Maturation pattern in some fiber tracts is still poorly understood due to extant disagreements on the structure of the tracts. Further, a thorough review and meta-analyses of human MRI and postmortem data cautions that gender effects in human brains are largely based on overall brain size and in the few instances that they appear they contribute only to 1% of variance in structure (Eliot et al., 2021). Therefore, when investigating gender-related differences in WM development and microstructure, it is important to consider differences in total brain size, and to take into account specificity of the method applied and age of the participants, since myelination rate at different developmental stages is different. Moreover, the comparison of the neuroimaging results with the post mortem findings may help to clarify the discrepancies across methods. Further research, especially longitudinal studies and meta-analyses that identify overarching patterns in the data, are needed to evaluate effects in WM between the two genders.

## Myelination and Cognitive Abilities in Common Fiber Tracts

White matter pathways, which connect distributed brain areas, play a fundamental role in the maintenance of higher-level cognitive functions and are pivotal for cognitive, motor, and behavioral performance. A DTI study by Johansen-Berg et al. (2007) found an association between bimanual coordination and characteristics of WM microstructure in the CC which connects the supplementary motor area and caudal cingulate motor area. Compared to an object recognition task (Baird et al., 2005), high FA in the splenium and genu of the CC were associated with shorter and longer reaction times, respectively. The task on object recognition was presented from unusual viewpoints, which required transduction of the information from the right parietal cortex to the left inferior parietal cortex. Since myelin sheath increases signal transduction and its formation continues during adulthood, brain myelination plays an important role in inhibitory control and executive functions in children and adolescents, thus underlying healthy maturation (Bartzokis and Altshuler, 2005).

Research suggests that greater axonal volume and myelination in particular brain regions, including the parietal cortical areas, left superior and posterior corona radiata, and body of the CC contribute to better performance on auditory working memory tasks, such as letter-number sequencing, by sustaining higher speed of information processing (Chung et al., 2018). Tamnes et al. (2013) suggest that myelination and maturation of synaptic connections in fronto-parietal brain networks during adolescence is implicated in greater specialization and processing efficiency, which, in turn, mediates the development of executive functions in children and adolescents. As shown in a DTI study by Tuch et al. (2005), the correlation between microstructural measurements of WM in the right projection and association pathways, such as the right optic radiation, right posterior thalamus, and right medial precuneus, with visual self-paced choice task performance, reflects the fundamental role of myelination in these regions for maintenance of visuospatial attention. Notably, microstructural characteristics of WM pathways, including FA and neurite density, show a positive correlation with mathematical performance in 13-year-olds (Collins et al., 2019). Myelination indices in the left superior corona radiata correlate with numerical operations and mathematical reasoning, whereas WM microstructure characteristics in the left ILF show associations with numerical operations specifically (van Eimeren et al., 2008).

Myelin is a principal component of WM and it accounts for approximately 50-60% of the WM dry weight (Snaidero and Simons, 2014). Stimulation of oligodendrocyte precursor cells results in their transformation to mature oligodendrocytes (Snaidero et al., 2014). Although the majority of tracts are myelinated by adulthood, several WM areas, such as those in prefrontal regions and optic radiation, reach peak of myelination between the third and fourth decades of life (Giedd et al., 2015; Turner, 2019). Some suggest that myelination is mainly driven by neuronal activity (i.e., generation and passage of action potentials along the axonal membrane), which can trigger an increase in axonal diameter (Costa et al., 2018). Once the axonal diameter exceeds 0.5 mcm (Nave and Werner, 2014) and the number of action potentials generated along the unmyelinated axonal membrane is sufficient to stimulate conversion of oligodendrocyte precursor cells to mature cells, oligodendrocytes wrap nearby axons by myelin layers (Snaidero et al., 2014).

In mature axons, fine tuning of myelination speed and modulation of signal transmission are controlled by changes in myelin thickness (Waxman, 1975; Campbell et al., 2018) and the length of myelinated axonal regions (Rushton, 1951; Brill et al., 1977), axonal diameter (Chéreau et al., 2017), and topographical organization of the nodes of Ranvier within the axons (Arancibia-Cárcamo et al., 2017). Particularly, along with the number of myelin layers and internode length, node organization to a large extent determines conduction speed along myelinated axons, and modulation of the lengths of the nodes of Ranvier serves an efficient mechanism to control signal conduction (Arancibia-Cárcamo et al., 2017).

It is proposed that myelination can be activity-dependent (i.e., depend on electrical activity of the axon and various molecular mediators synthesized and released in response to electrical events), and activity-independent (i.e., regulated by oligodendrocytes and independent from on the axonal electrical activity; De Faria et al., 2019 for review). An intriguing work by Bechler et al. (2018) discusses a “smart wiring” model of myelination, which includes two phases – intrinsic and adaptive, and suggests possible mechanisms by which active axons may become more myelinated and how brain circuits may be modified in response to learning and new experience. Bechler also discusses adaptive myelination, which is provided by several properties of oligodendrocytes. Oligodendrocytes in the central nervous system are generated from special progenitor cells, which actively divide throughout the whole life (Rivers et al., 2008; Hughes et al., 2013). Moreover, proliferation and differentiation of oligodendrocyte precursor cells was demonstrated to improve motor learning and learning new motor skills, which in turn, changes the structure of the WM containing many late-born oligodendrocytes (McKenzie et al., 2014). Overall, brain myelination appears to be adaptive, and it may change in response to learning and new experience that relates to fiber tracts that connect brain areas associated with various cognitive, affective and motor functions.

Therefore, research supports that increasing neuronal activity, which accompanies brain maturation and learning is the main driving force for dynamic myelination and regulation of network organization throughout lifespan (Hill et al., 2018; Hughes et al., 2018; Stedehouder et al., 2018). By modulating oligodendrocyte precursor cell proliferation and oligodendrocyte differentiation, increased neuronal activity ensures efficient signal transmission, fine-tuning of conduction speed, and the maintenance of circuit function. Myelin growth, in turn, promotes an additional increase in conduction velocity, thus strengthening the existing connections (Fields, 2015; Almeida and Lyons, 2017). This is consistent with developmental cognitive theory that proposes neuropsychological mechanisms that underlie these changes. Specifically, the Theory of Constructive Operators (Pascual-Leone, 1970; Arsalidou et al., 2019; Pascual-Leone and Johnson, 2021) proposes that operators (i.e., content-free general resources represented by functional-structural constrains) regulate schemes (i.e., information bearers represented by cell assemblies and networks) that produce a mental or action output when following the principle of schematic overdetermination of performance (i.e., following a winner-takes-all approach). This framework allows for ontogenetic, biological influences to drive maturation (i.e., relate more to operators) as well as experiential opportunity to drive learning (i.e., relate more to schemes). As we overview below, structure-function relations may be supported by mechanisms associated with biological maturation and experiential learning.

### Corpus Callosum

The largest fiber tract in the brain is the CC. It consists of more than 200 million axons and plays a key role in integration and interhemispheric transfer of information (Fitsiori et al., 2011). Malformation of the CC results in deficits in higher-level cognitive functions and social communication (Paul et al., 2014), as well as in a slower rate of cognitive processing, impaired language function (Banich and Brown, 2000; Paul et al., 2003), abstract reasoning, and concept formation (Brown and Paul, 2000). Incomplete myelination of the CC in 6–7-year-old children is associated with behavioral abnormalities and results in impaired transfer of complex visuomotor skills learned by the dominant hand from one hemisphere to another (Chicoine et al., 2000). The observation that myelination of the anterior portion of the CC may be induced by working memory training suggests plasticity of WM microstructure in this region (Takeuchi et al., 2010). Reduced integrity of the CC, in turn, may lead to working memory deficits. In particular, reduced integrity in the callosal subregions, which connect anterior and posterior parietal cortical areas, is associated with lower performance in verbal working memory tasks, and higher RD in the portion of the CC, which links anterior regions with posterior and temporal cortical areas, predicts lower performance in visuospatial working memory task (Treble et al., 2013). Interestingly, the length of the CC shows negative correlation with nonverbal abilities (Schmied et al., 2020). Together with the cingulum, the CC often shows correlations with functional measures in neurological groups (Forkel et al., 2020).

### Cingulum

A number of DTI studies show that the cingulum, especially its dorsal portion, is implicated in cognitive control and various executive functions, including shifting and inhibition (Kantarci et al., 2011; Bettcher et al., 2016), updating and working memory (Charlton et al., 2010; Kantarci et al., 2011; Bettcher et al., 2016), processing speed (Takahashi et al., 2010; Bettcher et al., 2016), and sustained attention (Takahashi et al., 2010). In particular, FA in the right cingulum correlates with task performance on the Continuous Performance Test, which measures sustained attention (Takahashi et al., 2010). MD in the bilateral cingulum shows gender integrations and is associated with internalizing behavior in children and adolescents, which is more pronounced in females (Andre et al., 2020). Interestingly, FA in the left cingulum, as well as in the left UF, shows strong age-behavior interaction (Andre et al., 2020). Microstructural characteristics in the posterior cingulum also demonstrate a strong association with language (category fluency and naming to confrontation) and visual-spatial functions, whereas FA in both posterior and anterior portions of the tract are implicated in attention, executive functions, and memory assessed by free recall retention task performance and auditory verbal learning test (Kantarci et al., 2011). The strongest evidence about the contribution of the cingulum to episodic and verbal memory functions are demonstrated in clinical samples, including Alzheimer’s disease (Lin et al., 2014; Kantarci et al., 2017), amnestic mild cognitive impairment (Lin et al., 2014; Yu et al., 2017), cerebral small vessel disease (van der Holst et al., 2013), and acute mild traumatic brain injury (Wu et al., 2010). Together with the AF and UF, the microstructural properties of the cingulum are often reported to be strongly associated with psychiatric symptoms (Forkel et al., 2020).

An intriguing article by Bathelt et al. (2019) describes the use of a data-driven community-clustering algorithm to analyze differences in WM microstructure in children and adolescents (mean age ∼11), which identified two subgroups characterized by a prominent difference in FA in bilateral cingulum. Critically, these two brain types differed in cognitive abilities with the higher FA group exhibiting better performance in working memory and long-term memory tasks, fluid intelligence, and vocabulary. Long fibers in the dorsal cingulum run in parallel with the long-range fibers in the SLF, which, in turn, extent more laterality to the AF in each hemisphere.

### Superior Longitudinal Fasciculus and Arcuate Fasciculus

Diffusion tensor imaging studies show that the right SLF is associated with various complex cognitive functions, including attention (Frye et al., 2010) and visuospatial abilities (Hoeft et al., 2007), whereas left SLF plays a fundamental role in language (Dick and Tremblay, 2012) and reading skills (Frye et al., 2010). In adolescents, bilateral SLF is involved in verbal working memory and verbal fluency (Peters et al., 2012). It is proposed that the SLF consists of five branches (Kamali et al., 2014). The SLF I is involved in the regulation of motor behavior and the initiation of motor activity. In addition, it was suggested to transfer somatosensory and kinesthetic information about location of body parts, including trunk and limbs, to the frontal regions of the brain (Schmahmann and Pandya, 2006). The SLF II sustains visual perception and it is a part of the circuit involved in visual awareness and the maintenance of attention. The SLF II fibers connecting the prefrontal cortex with the posterior parietal regions are implicated in regulation of the focusing of attention (Petrides and Pandya, 2002). The SLF III was found to convey higher-level somatosensory information and to be involved in gestural communication (Petrides and Pandya, 2002). The dorsal pathway through the lateral SLF supports articulatory function, phonological working memory (Duffau, 2019), perception and production of speech (Indefrey and Levelt, 2004; Hickok and Poeppel, 2007). The AF is implicated in intelligence, reasoning abilities (Lebel and Beaulieu, 2009), and language processing (Geschwind, 1970), playing a critical role in auditory to motor connection (Webb, 2017) and semantic and phonological processes associated with visual information (Zemmoura et al., 2015). The indicators of WM microstructure in the left SLF demonstrate a negative association with inhibitory control, whereas in the left AF they correlate with intelligence tests and attention (Urger et al., 2015). Therefore, findings from the neuroimaging studies demonstrate a critical role of the SLF and AF in neurophysiological abilities and cognitive function.

### Inferior Longitudinal Fasciculus

The ILF (Shinoura et al., 2007), together with the MdLF (Saur et al., 2008) and UF (Papagno et al., 2011), comprises the ventral stream of language processing and was found to sustain speech comprehension and general semantic processes (Rizio and Diaz, 2016). A proposed role of the ILF in semantic functionality (Mandonnet et al., 2007) is supported by neuroimaging data obtained in the study by Shin et al. (2019), which demonstrates the role of the ILF in language comprehension. The ILF connectivity pattern was also shown to be leftward-lateralized and greater fiber density and myelination in this region were found to be associated with better semantic processing. Moreover, the ILF is known to be implicated in object (Ortibus et al., 2012) and face (Taddei et al., 2012) recognition. Some earlier studies show that the peak of myelination in the ILF occurs at 11 years of age (Lebel et al., 2008), whereas recent findings demonstrate that the maturation of this fiber tract continues until age of 24 years (Lebel and Beaulieu, 2011; Lebel and Deoni, 2018). Notably, age has a great impact on lateral tracts in dorsal areas of the brain, including the SLF, whereas the inferior regions are less affected by aging (Sullivan et al., 2010). The ILF, together with the AF, demonstrates strong functional correlations with cognitive measure in healthy individuals (Forkel et al., 2020).

### Inferior Fronto-Occipital Fasciculus

Diffusion MRI tractography studies suggest the role of the IFOF in semantic and visual processing and attention (Catani and Thiebaut de Schotten, 2008; Leng et al., 2016). In particular, bilateral IFOF (especially in the left frontal and right occipital parts) is implicated in the maintenance of executive functions, whereas the parietal and insular parts of the left IFOF are involved in alerting (Leng et al., 2016). Higher FA in the IFOF predicts better reading scores (Lebel et al., 2013), and FA in the left IFOF is related to better orthographic processing (Vandermosten et al., 2012). In addition, the IFOF plays a crucial role in multimodal semantic processes, including naming and non-verbal semantic associations (Gil-Robles et al., 2013; Han et al., 2013; Moritz-Gasser et al., 2013; Rollans et al., 2017), constituting a bilateral network, which sustains non-verbal semantic system (Herbet et al., 2017). Moreover, microstructural properties of the IFOF predict object working memory task performance and therefore underlie nonspatial working memory processing (Walsh et al., 2011). However, the anatomical features of the IFOF are still a matter of discussion and there is still no consensus regarding the mechanisms and the degree to which anatomical and microstructural properties of this bundle can vary in healthy brains.

### Uncinate Fasciculus

A DTI study by Sato et al. (2012) demonstrates the association between DTI metrics in the UF and visual memory task performance. Studies of mild cognitive impairment and psychopathology support a critical role of the UF in higher-order cognitive functions, including attention, spatial memory, and emotion recognition (Fujie et al., 2008; Hiyoshi-Taniguchi et al., 2015; Olson et al., 2015; Singh et al., 2016). The involvement of the UF in visual associative learning was found in the DTI study by Thomas et al. (2012), who demonstrated a strong positive association between learning rate and microstructural properties of the left UF, suggesting a critical role of the UF in information retrieval. Other DTI studies show strong relations among microstructural characteristics of this WM tract in the left (McDonald et al., 2008; Niogi et al., 2008) and right (Fink et al., 2010) hemispheres and auditory-verbal processes, but not visual memory in adults (McDonald et al., 2008; Niogi et al., 2008), children, and adolescents (Mabbott et al., 2009). The left and right UF may sustain different neurophysiological processes. As such, Thomas et al. (2015) suggest that the left UF contributes to rapid learning of conditional visual-visual associations, whereas the right UF may mediate immediate retrieval of these associations.

### Middle Longitudinal Fasciculus

Microdissection data on spatial organization of the MdLF point at its role in auditory comprehension as a part of the dorsal auditory stream (Wang et al., 2013). Saur et al. (2008, 2010) found that MdLF, together with the extreme capsule, connects cortical regions involved in comprehension of meaningful compared to non-meaningful sentences. Although the functional role of the MdLF is still unknown, some suggest that its anatomy relates to a role in language (Schmahmann and Pandya, 2006; Makris et al., 2009). However, electrical stimulation and resection of this fiber tract in the language dominant hemisphere of a patient with brain tumor did not cause language deficits (De Witt Hamer et al., 2011). Further, as evidence by DTI studies, FA in the MdLF, as well as the tract volume, does not display hemispheric asymmetry, as expected for language-related tracts (De Witt Hamer et al., 2011; Wang et al., 2013). More research is needed to understand the functional role of this fascinating fiber tract.

### Frontal Aslant Tract

Neurosurgical studies suggest the role of FAT in speech planning (Chernoff et al., 2018) and initiation (Fujii et al., 2015), and it is hypothesized to subserve a link between sentence planning and lexical access (Chernoff et al., 2018). Moreover, FAT was recently identified as a part of a motor language stream involved in speech production (Dick et al., 2013). The role of FAT in language can be inferred from its leftward lateralization in right-handed individuals, similar to other language-related fiber systems (Catani et al., 2012). Interestingly, intraoperative direct current stimulation of the left FAT leads to shuttering (Kemerdere et al., 2016) and speech deficits (Vassal et al., 2014). Damage to this fiber tract leads to verbal fluency, but not grammar deficits in primary progressive aphasia (Catani et al., 2013). Notably, DTI studies suggest that individual microstructural characteristics of FAT maintain kinematics and visuomotor processes, and are involved in visually guided hand movements, such as grasping (Budisavljevic et al., 2017). In older adults, DTI characteristics of FAT, together with the left SLF, predict working memory performance and are hypothesized to be involved in the maintenance, attending, and manipulation of language-related information (Rizio and Diaz, 2016; Varriano et al., 2018). Due to limited data on the association between FAT microstructure and cognitive function, the role of this fiber pathway is mainly evidenced from studies of functional role of the cortical regions it connects. As such, the role of the right IFG and pre-SMA/SMA in inhibitory control may indicate the involvement of the FAT connecting these regions in executive functions (Dick et al., 2019). There are many questions that remain to be answered on the intricate relation of mental function to structural properties of FAT that link psychology, biology and neuroscience.

## Conclusion

Biological development is associated with maturation of WM pathways which connect distant and proximal brain regions and are critical components of cognitive processing. Our review suggests that myelination follows a complex developmental trajectory that may vary by age, fiber tract and hemisphere. Effects of gender were also identified, although differences may be confounded by methodological factors (e.g., not controlling for brain volume) or social and learning opportunities. Although different studies use various methodological approaches and it is a challenge to aggregate data, future studies should examine overarching pattern across studies using meta-analytical approaches. The study of the individual differences in cerebral WM myelination is very important for developmental biology and neuroscience. Practically, understanding how and when myelination changes and its relation to cognitive performance can inform education practice and clinical interventions.

## Author Contributions

IB and MA conceptualized the project and revised and finalized the manuscript. IB wrote the first draft. MA edited versions of the manuscript. Both authors contributed to the article and approved the submitted version.

## Conflict of Interest

The authors declare that the research was conducted in the absence of any commercial or financial relationships that could be construed as a potential conflict of interest.
